# What drives preservice teachers’ use of generative AI as instructional media? A structural and configurational analysis

**DOI:** 10.3389/frai.2026.1853130

**Published:** 2026-07-09

**Authors:** Francis Arthur, Francis Obeng Gyedu, Emmanuel Quayson, Silas Afutu Quaye, Eric Boateng, Mark Inkoom, Sharon Abam Nortey, Dorcas Frempong

**Affiliations:** 1 Faculty of Humanities and Social Sciences Education, Department of Business and Social Sciences Education, University of Cape Coast, Cape Coast, Ghana; 2 Institute of Educational Planning and Administration, University of Cape Coast, Cape Coast, Ghana; 3 Institute for Oil and Gas Studies, University of Cape Coast, Cape Coast, Ghana; 4 Faculty of Educational Foundations, Department of Psychology, University of Cape Coast, Cape Coast, Ghana

**Keywords:** behavioural intention, fsQCA, generative artificial intelligence, instructional media, PLS-SEM, preservice teachers, UTAUT2

## Abstract

**Introduction:**

The accelerating diffusion of Generative AI (GenAI) in education has sparked interest in understanding how preservice teachers adopt it as instructional media. Drawing on the “Unified Theory of Acceptance and Use of Technology 2 (UTAUT2)” as a guided framework, this study examined cognitive, motivational, and contextual drivers of Generative AI use among preservice teachers in Ghana.

**Methods:**

The descriptive cross-sectional survey design was used and data were collected from 783 preservice teachers using validated questionnaires. The study used the “Partial Least Squares Structural Equation Modelling (PLS-SEM) and Fuzzy Set Qualitative Comparative Analysis (fsQCA)” to analyse the data.

**Results and discussion:**

The structural model revealed that behavioural intention strongly predicted GenAI use. Performance expectancy, perceived learning opportunity, perceived trust, and social impact significantly influenced intended behaviour. However, the conditions for facilitation, perceived learning opportunity, and perceived trust directly predicted actual use. Together, the model was able to explain 64.7% of the variance in behavioural intention and 68.8% in GenAI use, both using strong predictive relevance. FsQCA results revealed multiple sufficient configurations leading to high behavioural intention and GenAI use. This emphasises that diverse combinations of cognitive, institutional, and affective factors can drive adoption. The findings further highlight that perceived trust and learning opportunities are central to preservice teachers’ engagement with GenAI. Also, behavioural intention and structural support remain necessary for sustained use. The study offers both theoretical and practical contributions for embedding GenAI literacy and innovative teaching techniques into teacher education programmes.

## 1 Introduction

The roll-out of Generative AI (GenAI) technologies into educational contexts has emerged as a transformative force in contemporary teacher preparation programs worldwide. As educational institutions increasingly acknowledge the capacity of AI-driven tools to improve instructional practices, understanding how preservice teachers adopt and utilize these technologies becomes critical for effective teacher education (Nyaaba et al., 2024). The swift advancement of GenAI applications, especially large language models like ChatGPT, has created unprecedented opportunities for educational innovation while simultaneously raising important questions about their appropriate integration into pedagogical practices.

Pre-service teacher education represents a key juncture within professional development of future educators, where foundational pedagogical knowledge, technological competencies, and instructional practices are established (Alahmad et al., 2021; Tomczyk, 2024). The effective incorporation of emerging technologies during this formative period can significantly influence how new teachers approach instruction throughout their careers (Rowston et al., 2022). However, the uptake and effective use of Generative AI technologies among pre-service teachers involves complex interactions between technological, pedagogical, and contextual factors that require systematic investigation (Spasopoulos et al., 2025).

Research across multiple international contexts demonstrates that preservice teachers’ adoption of GenAI as an instructional medium varies by complex interactions of technological, psychological, and contextual variables. Studies from the United States, China, Australia, and Ireland have consistently identified that how useful, easy to use, and confident student teachers feel about GenAI significantly shapes their decision to adopt it Barbieri and Nguyen (2025), Hsu et al. (2024), Liu et al. (2025), and Zheng et al. (2025). Arthur et al. (2025) indicated that behavioural intention toward GenAI adoption was meaningfully influenced by performance expectancy, hedonic motivation, facilitating conditions, personal innovativeness, perceived learning opportunities, and satisfaction. Furthermore, versions of the Technology Acceptance Model (TAM) and the Unified Theory of Acceptance and Use of Technology (UTAUT) have proven effective in explaining both behavioural intention and actual usage patterns within a variety of educational settings (Liu et al., 2025; Unal and Uzun, 2020; Wang et al., 2024; Iqbal et al., 2025).

The potential benefits of GenAI integration within pre-service teacher education extend beyond mere technological adoption. Emerging evidence suggests that GenAI-assisted instruction correlates with meaningful academic and professional outcomes, including reduced praxis shock, lower stress levels, improved confidence, and enhanced academic achievement (Barbieri and Nguyen, 2025; Iqbal et al., 2025). These benefits appear to be mediated through cognitive mechanisms such as shared metacognition and cognitive offloading, suggesting that GenAI tools may fundamentally alter how pre-service teachers process information and approach instructional tasks (Iqbal et al., 2025).

However, the acceptance and utilization patterns of GenAI technologies are not uniform across different cultural and educational contexts. Demographic factors including age, gender, and academic level, as well as attitudinal elements including innovation consciousness and AI knowledge, significantly influence usage patterns (Nyaaba et al., 2024; Merzifonluoglu and Gunes, 2025). This contextual variability highlights the significance of carrying out region-specific research to understand how local factors shape GenAI adoption in teacher education.

Pre-service teacher education in Ghana is delivered through a network of higher education institutions comprising public and private colleges of education and universities. Following the 2018 teacher education reform, colleges of education transitioned to a four-year Bachelor of Education (B. Ed.) programme as the standard qualification for basic school teachers. The universities continue to provide undergraduate and postgraduate teacher education programmes (GTEC, 2025). The reform was guided by the National Teacher Education Curriculum Framework and national teacher standards aimed at improving teacher quality among pre-service teachers (Asare and Nti, 2014; UNESCO, 2023). National policy initiatives, including the Education Strategic Plan (2018–2030), ICT in Education Policy, and the ICT Competency Framework for Teachers, further emphasize the integration of digital technologies and emerging AI-related competencies into teacher preparation (Adjei et al., 2026; UNESCO, 2023). However, recent studies find that Colleges of Education still struggle with limited technology use due to infrastructure shortages and insufficient training (Adjei et al., 2026).

Despite the growing international recognition of GenAI as a revolutionary instructional medium in teacher education, significant gaps persist in understanding the complex mechanisms underlying preservice teachers’ acceptance and application of these technologies, particularly within educational institutions in sub-Saharan African. While research from developed countries has consistently identified perceived usefulness, ease of use, and self-efficacy as significant indicators of prediction of GenAI use (Liu et al., 2025; Zheng et al., 2025; Barbieri and Nguyen, 2025), the limited research from Ghana reveals that demographic characteristics, including age and year of study significantly impact the frequency of usage, while gender shows no significant effect (Nyaaba et al., 2024). However, this foundational understanding fails to capture the configurational complexity of GenAI adoption, where multiple combinations of technological, pedagogical, and contextual factors may lead to equivalent outcomes through different pathways. The predominant reliance on traditional linear modelling approaches in existing studies (Wang et al., 2024; Iqbal et al., 2025) inadequately addresses the nuanced, context-dependent nature of technology adoption in resource-constrained educational environments.

Furthermore, the methodological limitations found in current study approaches restrict a comprehensive understanding of how cultural, infrastructural, and pedagogical contexts unique to developing countries influence GenAI integration patterns among preservice teachers. While studies have documented benefits such as reduced praxis shock, enhanced confidence, and improved learning outcomes through mechanisms like shared thinking and the offloading of cognitive tasks (Barbieri and Nguyen, 2025; Iqbal et al., 2025), these findings emerge primarily from Western educational contexts and may not adequately reflect the realities of teacher preparation programs in sub-Saharan Africa. The absence of integrated analytical frameworks that can simultaneously examine both variance-based relationships and configurational patterns limits the development of contextually appropriate strategies for supporting GenAI adoption.

This limitation necessitates the application of advanced analytical techniques that can accommodate multiple causal pathways and configurational thinking. In response to this need, the present study adopts a combined approach using “Partial Least Squares Structural Equation Modelling (PLS-SEM) and Fuzzy-Set Qualitative Comparative Analysis (fsQCA)” to investigate how student teachers in Ghana use GenAI as a teaching tool. The methodological integration for this study helps the identification of structural relationships between key constructs and the exploration of sufficient configurations of conditions that contribute to successful GenAI adoption and use, building upon the foundational work established by previous researchers in this rapidly evolving field.

## 2 Theoretical foundation and hypotheses development

### 2.1 Theoretical foundation

The rapid diffusion of generative AI (GenAI) technologies, models and systems that produce text, images, and other pedagogical artifacts on demand has produced new opportunities and challenges for teacher education (Giannakos et al., 2025). Preservice teachers can potentially leverage GenAI as an instructional medium to accelerate lesson planning, diversify learning materials, and scaffold student-centered activities. Yet adoption is facilitated by a constellation of cognitive, social, institutional, motivational, as well as trust-related variables (Anwar et al., 2025). To explain what drives preservice teachers’ behavioural intention (BI) and actual use (Use) of GenAI for instruction, this study situates its model in the UTAUT2 (Venkatesh et al., 2016). It also integrates complementary perspectives from TAM, Innovation Diffusion Theory (IDT), trust-based perspectives on AI adoption, and constructivist and self-determination approaches to learning. UTAUT2 is especially appropriate because it combines performance- and effort-based beliefs with hedonic and habitual influences and explicitly models favourable conditions and social impact. All of which remain important in educational settings where infrastructure, enjoyment, and habit shape technology uptake.

Furthermore, UTAUT2 identifies the primary factors that drive behavioural intention and actual use, namely performance expectancy (PE), effort expectancy (EE), social influence (SI), facilitating conditions (FC), hedonic motivation (HM), and habit (HT) (Venkatesh et al., 2016). In teacher education, PE (the belief that GenAI will improve instructional effectiveness) and EE (the perceived ease of using GenAI) anchor teachers’ cost–benefit calculus. Also, SI captures the normative pressures and modelling from lecturers, mentors, and peers. FC denotes institutional resources (devices, connectivity, training, policy) that enable translation of intention into practice. Hedonic motivation and habit are particularly relevant for GenAI because its generative affordances can provide novelty and enjoyment while repeated use of digital tools creates routinized behaviours that shortcut deliberative decision-making. Empirical meta- and field studies in educational technology confirm UTAUT2’s predictive power for teachers’ intentions and use of novel ICTs, including emergent AI tools.

### 2.2 Hypotheses development

#### 2.2.1 Behavioural intention (BI) and actual use of GenAI (USE)

In most behavioural theories such as the Theory of Planned Behaviour (TPB) (Ajzen, 1991) and the UTAUT2 (Venkatesh et al., 2016), behavioural intention is treated as a key proximal driver of actual behaviour (Use). In educational technology adoption, teachers’ willingness to use a system strongly predicts whether they eventually incorporate it into their teaching (Dwivedi et al., 2021; Teo et al., 2018). Studies have consistently supported this linkage. For example, Abreh et al. (2025) established that the intention of Ghanaian STEM students to learn AI led to clear and measurable engagement behaviours. Similarly, Arthur et al. (2025) showed that higher education students’ behavioural intention toward ChatGPT significantly predicted actual use for learning. Basri (2024) also confirmed that behavioural intention mediated teachers’ use of AI-enabled learning management systems. However, some research suggests the intention-behaviour gap can arise under certain conditions. For example, if institutional support is lacking, intention may not fully translate into use. Bayaga and du Plessis (2024) found that facilitating conditions moderated this relationship, such that without adequate support, intention did not lead to actual use. Therefore, preservice teachers’ actual use of GenAI as instructional media will likely depend on the strength of their prior intention, while noting that actual adoption also requires supportive contextual factors.

*H_1_:* Preservice teachers’ behavioural intention to use GenAI positively predicts their actual use of GenAI as an instructional media.

#### 2.2.2 Effort expectancy (EE) and Behavioural intention (BI)

Effort expectancy reflects the level to which users consider a technology simple and not mentally taxing to use (Oyewole, 2018). When GenAI tools like ChatGPT, Google Gemini, or Microsoft Copilot are seen as intuitive and requiring little cognitive effort, teachers are able to form positive intentions to use them. In teacher education contexts, several studies confirm this relationship. Teo et al. (2018) found that ease-of-use perceptions were critical to preservice teachers’ adoption of mobile learning tools. Similarly, Nguyen et al. (2024) reported that AI tutors’ simplicity increased university students’ acceptance in online learning environments. Abreh et al. (2025) noted that effort expectancy showed a significant antecedent of Artificial Intelligence learning intention among STEM students, particularly for learners with limited prior exposure. Other studies, such as Ayanwale (2023) in Lesotho showed that the simpler the AI interface, the greater the likelihood of continued engagement. But some research finds the direct effect of EE on intention to be weak or non-significant. For instance, Bervell et al. (2022) and Zacharis and Nikolopoulou (2022) reported no significant relationship between effort expectancy and behavioural intention in teaching contexts. This suggests that when users already expect some level of support or when other factors (like performance expectancy) dominate, the ease-of-use effect can diminish. Nevertheless, in demanding pedagogical settings, tools perceived as user-friendly are likely to reduce cognitive barriers. We therefore posit:

*H_2_:* Higher effort expectancy positively influences preservice teachers’ behavioural intention to use GenAI.

#### 2.2.3 Facilitating conditions (FC)

Facilitating conditions capture user” perception of whether the required institutional, technological, and organisational backing exists to help them use a technology effectively (Venkatesh et al., 2016). In teacher education, this consists of availability of computers, reliable internet, training, and school-level encouragement. Empirical research has consistently confirmed FC’s importance. Abreh et al. (2025) showed that facilitating conditions strongly predicted Ghanaian students’ AI learning intention, highlighting how institutional resources enable digital participation. Teo et al. (2018) observed similar patterns in ICT use among preservice teachers in Singapore, while Ng et al. (2023) reported that training and infrastructural support determined teachers’ integration of AI-based assessment tools. Cambra-Fierro et al. (2025) also showed that university lecturers’ use of ChatGPT depended heavily on institutional guidelines and technological readiness. On the other hand, some research reports weak or indirect effects of FC. Bervell et al. (2022) found no direct effect of FC on teachers’ intentions, and Riady et al. (2022) observed an insignificant correlation between FC and intention in a large teacher sample. This mixed evidence suggests that while structural support often matters, its impact may depend on context and mediation by other factors. In our study, we examine both intention and use:

*H_3_:* Stronger facilitating conditions positively influence behavioural intention to use GenAI.

*H_4_:* Stronger facilitating conditions positively predict actual use of GenAI.

#### 2.2.4 Hedonic motivation (HM)

Hedonic motivation reflects the extent to which using a technology is seen as enjoyable and pleasurable by its users (Oluwajana et al., 2019). For GenAI, hedonic motivation arises from creative engagement such as auto-generating lesson plans, quizzes, or teaching visuals, which enhances teaching enjoyment. Research shows that enjoyment significantly drives technology acceptance. Salifu et al. (2025), Wei et al. (2024) and Sallam et al. (2025) found that the creativity and pleasure of using AI increased teachers’ adoption intentions, and Zawacki-Richter et al. (2024) and Zhang et al. (2025) argue AI’s novelty generates sustained engagement. It should be noted, however, that some studies suggest intrinsic enjoyment plays a smaller role in formal educational settings, where utilitarian outcomes often dominate. Even so, because GenAI offers novel interactive experiences, we expect hedonic motivation to be positively related to intention in our context.

*H_5_:* Higher hedonic motivation positively influences preservice teachers’ behavioural intention to use GenAI.

#### 2.2.5 Habit (HT)

Habit captures the level to which a behaviour becomes automatic due to practice and repetition (Kumar et al., 2020). Teachers who routinely use digital tools (e.g., LMSs, plagiarism checkers, Google Classroom) may find it natural to incorporate GenAI into their instructional practices. Empirical evidence is mixed. Studies like Jang et al. (2021) and Mangena (2023) report habitual technology use predicts both intention and actual use among educators, and Guan et al. (2025) found preservice teachers with tech habits adopted AI pedagogy earlier. UTAUT2 theorizes habit has a direct effect on use beyond intention (Venkatesh et al., 2012). However, since GenAI is new, preservice teachers may not yet have formed habits with it. Habit may thus influence actual use more than initial intention in this case. We hypothesize positive effects following the theory:

*H_6_:* Stronger habit positively influences behavioural intention to use GenAI.

*H_7_:* Stronger habit positively predicts actual use of GenAI.

#### 2.2.6 Performance expectancy (PE)

Performance expectancy or perceived usefulness reflects the level to which individuals believe that a technology will help them perform their work more effectively (Sair and Danish, 2018). In education, this means believing GenAI will improve instructional delivery, feedback, and student engagement. PE is often the strongest predictor of intention in UTAUT-based studies (Venkatesh et al., 2016). Prior studies in similar domains reinforce this. Islamoglu et al. (2021) found usefulness predicted mobile tech adoption by preservice teachers, and Nguyen et al. (2024), Saha et al. (2025), and Abreh et al. (2025) each report that higher perceived usefulness increases students’ AI learning intention. Arthur et al. (2025) similarly confirmed that PE strongly predicted ChatGPT use in higher education. Notably, Abreh et al. (2025) also observed that if enabling conditions are lacking, high PE can fail to materialize into adoption. In their case, perceived usefulness even negatively affected intention when support was insufficient. This suggests PE’s effect may be contingent on context. Nonetheless, we expect the basic relationship:

*H_8_:* Higher performance expectancy positively influences preservice teachers’ behavioural intention to use GenAI.

#### 2.2.7 Personal innovativeness (PI)

According to Innovation Diffusion Theory (Rogers, 2003), individuals differ in their willingness to adopt innovations. Personal innovativeness is an individual trait reflecting openness to trying new technologies (Nov and Ye, 2008). In teacher education, innovative preservice teachers are more willing to explore new technologies and adapt them in instructional contexts. Empirical studies highlight PI’s role in technology acceptance. Kim (2025) showed that teachers with stronger innovativeness exhibited the likelihood to adopt VR and artificial intelligence applications. Chai et al. (2021) demonstrated that personal innovativeness predicted AI learning intention among Chinese secondary students. Prilop et al. (2025) similarly noted that curiosity and early experimentation characterized teachers who integrated GenAI into lessons. In some cases when organizational adoption is driven by policy, PI’s effect may be smaller, but overall, we expect a positive relationship in this exploratory context:

*H_9_:* Greater personal innovativeness positively influences behavioural intention to use GenAI.

#### 2.2.8 Perceived learning opportunity (PLO)

Grounded in constructivist and self-determination frameworks (Prilop et al., 2025), perceived learning opportunity reflects preservice teachers’ belief that GenAI promotes active learning, creativity, and differentiation. When teachers perceive GenAI as enriching student engagement or fostering higher-order thinking, they become more motivated to integrate it. Chai et al. (2021) and Ji et al. (2024) showed that perceived learning utility and relevance significantly increased AI learning motivation. Abreh et al. (2025) revealed that when STEM students perceived AI to be career-relevant and beneficial to learning, their adoption intention rose sharply. Similarly, Meng et al. (2025) observed that student teachers who saw AI as a pedagogical asset were more likely to apply it in practice. Thus, we hypothesize:

*H_10_:* Stronger belief that GenAI enhances student learning positively influences behavioural intention to use it.

*H_11_:* Stronger perceived learning opportunity positively predicts actual use of GenAI.

#### 2.2.9 Perceived trust (PT)

Because GenAI involves autonomous content generation, trust is crucial for its adoption. Perceived trust refers to belief in the technology’s accuracy, dependability, fairness, and ethical operation (Gefen et al., 2003). If teachers doubt GenAI’s transparency or data security, they may resist integration. Empirical studies underscore this point. Deng et al. (2025) found that perceived AI trustworthiness increased employee adoption, while AI anxiety reduced it. Abreh et al. (2025) similarly noted AI anxiety significantly and negatively predicted learning intention, implying that higher trust counteracts apprehension. Abdulmuhsin et al. (2025) showed that AI trust enhances educators’ willingness to use adaptive assessment tools, while Batubara et al. (2025) observed that ethical confidence in AI predictions encouraged teachers to integrate AI into pedagogy. We therefore hypothesize:

*H_12_:* Higher perceived trust in GenAI positively influences behavioural intention to use it.

*H_13_:* Higher perceived trust positively predicts actual use of GenAI.

#### 2.2.10 Social influence (SI)

Social influence describes the perceived pressure exerted from important referents mentors, peers, and supervisors, to perform a behaviour (Al-Adwan et al., 2024). Within teacher education, modelling by university lecturers or recommendations by mentors often shape preservice teachers’ adoption of digital innovations. Many studies report SI as a strong determinant: for example, Abreh et al. (2025) showed subjective norms powerfully predicted Ghanaian students’ AI learning intention, and Owan et al. (2025) found peer and instructor endorsement significantly affected ChatGPT use. Milutinović (2022) similarly found that lecturers’ and colleagues’ attitudes strongly influenced preservice teachers’ intentions. However, some studies have reported varying or even negative effects of SI. Suki and Suki (2017) found no significant direct effect of subjective norm on teachers’ intentions, and Bandoh et al. (2024) and Cimperman et al. (2016) observed that social influence could negatively affect intention. This possibly reflect cases where peer skepticism deters use. In sum, social cues may either encourage or discourage AI adoption depending on context. We thus propose:

*H_14_:* Stronger social influence positively influences preservice teachers’ behavioural intention to use GenAI.

#### 2.2.11 Research model

**Figure 1:**
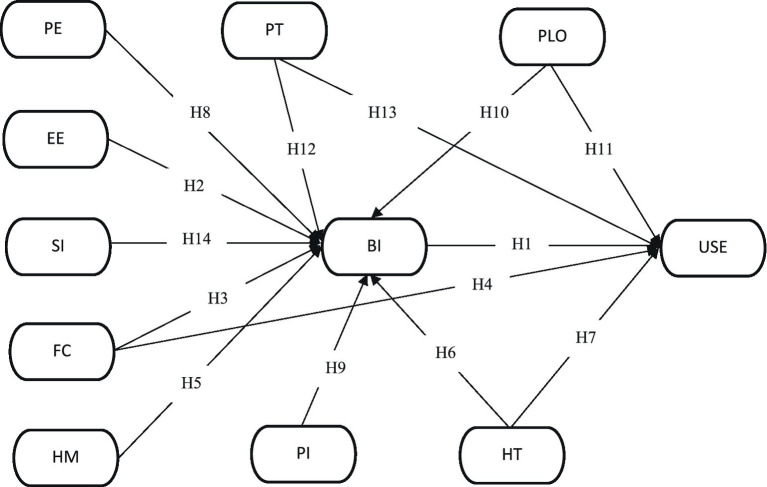
Research model. Source: Authors’ work. PE, performance expectancy; PT, perceived trust; EE, effort expectancy; SI, social influence; FC, facilitating condition; HM, hedonic motivation; PI; personal innovativeness; HT, habit; BI, behavioural intention; PLO, perceived learning opportunity; USE, use of GenAI.

Figure 1 shows the conceptual model of the study.

## 3 Methods

### 3.1 Study design and sample

This research is based on a positivist philosophy and applies a quantitative research approach. The study examined GenAI use as an instructional media among preservice teachers in Ghana. The study utilized a descriptive cross-sectional survey design. The design was suitable as it enables researchers to capture and measure variables within a specific period of time. It also helps identify patterns and relationships among variables within a large sample (Creswell and Creswell, 2017; Leavy, 2022). In addition, the descriptive cross-sectional survey design is used in quantitative studies where variables are observed in their natural setting. With this design, the researcher does not manipulate the variables being studied. The target population comprised preservice teachers in Ghana. These pre-service teachers are enrolled in Ghanaian teacher education institutions operating within the Bachelor of Education framework. The institutions integrate pedagogical theory, practicum experiences, and technology-supported instruction as part of teacher preparation. Participants were informed that the research examined how preservice teachers use GenAI as an instructional medium.

The study made use of the purposive sampling technique to involve 783 preservice teachers from the University of Cape Coast (UCC), Ghana. UCC was selected because it is one of the leading teacher education universities in Ghana and plays a central role in teacher preparation within the country. The university has a large and diverse population of preservice teachers drawn from different academic programmes and socio-cultural backgrounds. This makes it an appropriate institutional setting for examining technology adoption among future teachers. In addition, the university actively integrates technology-supported instruction and digital learning practices into teacher education. This provides participants with relevant exposure to emerging educational technologies such as Generative AI. The purposive sampling technique was chosen because it enabled the researchers to select preservice teachers who are knowledgeable about pedagogical contexts and issues pertaining to GenAI (Etikan et al., 2016). This made it feasible and efficient approach to collect data from participants whose insight would most directly inform the research hypothesis (Taherdoost, 2016).

### 3.2 Data collection procedure

The study gathered information using structured questionnaires distributed to the selected preservice teachers. Before the data collection process began, research assistants received training to support and manage the data collection activities. The investigators made sure they understood the intricacies of the survey instrument, and research ethics, and asked permission from the preservice teachers to get their consent before disseminating it. To improve the data-gathering procedure, the researchers had direct interactions with all preservice teachers in Ghana. The questionnaire took each respondent approximately 40 to 45 min to complete, giving them the time to think through and offer a considered response. This is because the study examined multiple latent constructs associated with Generative AI adoption, each measured using validated multi-item scales adapted from previous studies. The length of the instrument was therefore necessary to ensure adequate construct coverage, reliability, and measurement precision. To minimize response fatigue, the questionnaire was carefully structured using clear wording, logically sequenced sections, and concise item statements. Participants were also informed in advance about the estimated completion time and were allowed to complete the questionnaire at their own pace. Furthermore, the researchers carefully reviewed the completed questionnaires for completeness and correctness after collecting them.

### 3.3 Measures

This study explored preservice teachers’ use of GenAI as an instructional media in Ghana, using established instruments previously applied in studies on GenAI use in educational research (Foroughi et al., 2023; Mohd Rahim et al., 2022; Steil et al., 2020). Participants rated each section of the instrument through a five-point Likert scale, where 1 represented strongly disagree and 5 represented strongly agree. This scale, as noted by Buttle (1996), balances response quality with response rate, yielding high-quality data. The measurement items for human inventiveness and perceived learning opportunities were adapted from previous studies conducted by Strzelecki (2023), Foroughi et al. (2023), and Steil et al. (2020). Furthermore, the study’s constructs were drawn from earlier research by Foroughi et al. (2023), Mohd Rahim et al. (2022), Strzelecki (2023), and Venkatesh et al. (2012). These constructs include use behaviour, behavioural intention, hedonic motivation, habit, social influence and facilitating conditions. A panel of academics verified content validity.

### 3.4 Data analysis

Prior to conducting the analysis, data screening procedures were undertaken in order to eliminate duplicate entries and address missing or incomplete responses, ensuring the integrity of the dataset. This study analysed the associations among several casual as well as consequent variables using two analytical techniques. The first method was Partial Least Squares Structural Equation Modeling (PLS-SEM) implemented in SmartPLS 3.2.9 (Ringle et al., 2015). The second method was Fuzzy-Set Qualitative Comparative Analysis (fsQCA) conducted with version 4 software (Pappas and Woodside, 2021). The use of PLS-SEM was considered a suitable analytical tool due to its strength in dealing with complex models that contain multiple constructs and its effectiveness with small to moderately sized samples (Hair et al., 2017). Analysis for the study started with a review of the measurement model to confirm the reliability and validity of the constructs. Once this was done, attention shifted to the structural model to explore the proposed links between the study variables. Complementing this technique, fsQCA was employed to uncover distinct causal pathways leading to the outcome variable. This method explains individuals’ intention to participate in AI learning. It also provided a configurational explanation of the factors influencing this behaviour (Kumar et al., 2022; Geremew et al., 2024).

### 3.5 Ethical considerations

The research followed the ethical standards stated in the Helsinki Declaration for studies that involve human participants. Ethical approval for the study was obtained from the Institutional Review Board of the University of Cape Coast, Ghana (No. UCCIRB/CES/2025/26RV). Upholding participants’ rights and confidentiality was a central ethical consideration, and the researchers adopted rigorous procedures to safeguard these principles. Each participant received clear and comprehensive information about what the study sought achieve and were told that participation was completely voluntary. They were also free to withdraw from the study at any stage without incurring any form of penalty or forfeiting any entitlements. Strict anonymity was ensured by omitting identifiable details and maintaining the confidentiality of all sensitive data. The researchers prioritized the minimization of harm and discomfort, taking deliberate steps to prevent any potential adverse effects during the research process. Their unwavering commitment to ethical transparency and academic integrity was evident in all phases of the study. Moreover, every procedure was executed in alignment with institutional ethics protocols and legal requirements, thereby reinforcing the study’s credibility and ensuring respect for participants’ dignity, autonomy, and welfare.

## 4 Results

### 4.1 Normality results

An examination of skewness and kurtosis across 11 variables based on a sample of 783 participants showed that the data were not normally distributed. The results indicate that all univariate skewness and kurtosis values fall within permissible thresholds (±2 for skewness and ±10 for kurtosis), confirming univariate normality. However, Mardia’s multivariate kurtosis and skewness were significant (*p* < 0.001), indicating a deviation from multivariate normality. Owing to this violation, PLS-SEM was used for the analysis, given its suitability for handling data that are not normally distributed.

### 4.2 Common method bias

In order to detect common method bias (CMB), the researchers employed Variance Inflation Factors (VIF) alongside Harman’s single-factor test. The outcome revealed that a single factor contributed 22.09% of the total variance, staying well below the 50% limit proposed by Aguirre-Urreta and Hu (2019). These results suggest that the study’s findings are unlikely to be seriously affected by common method bias (Podsakoff et al., 2003). Furthermore, VIF values across all constructs ranged between 2.216 and 4.388, remaining well under the recommended threshold of 5 outlined by Kalnins and Praitis Hill (2023). This result shows that multicollinearity is not present in the data, and CMB is also not a concern. These results demonstrate that the dataset is reliable and the measurement model is sound.

### 4.3 Measurement model

All constructs in the measurement model successfully met the established benchmarks for reliability, internal consistency, and convergent validity. Item loadings were largely above the 0.70 cut-off, which indicates that each item adequately represents its corresponding construct (Hair et al., 2022). As shown in Table 1, Cronbach’s Alpha (*α*) values across all constructs surpassed the minimum acceptable threshold of 0.70, providing strong evidence of internal consistency (Hair et al., 2019; Hair et al., 2022).

**Table 1:** Construct reliability and validity.

Constructs	Items	Factor loadings	CA (α)	CR (rho_a)	CR (rho_c)	AVE
BI	BI1	0.839	0.895	0.896	0.927	0.760
	BI2	0.892				
	BI3	0.879				
	BI4	0.877				
EE	EE1	0.872	0.943	0.943	0.956	0.815
	EE2	0.917				
	EE3	0.916				
	EE4	0.900				
	EE5	0.907				
FC	FC1	0.870	0.906	0.908	0.934	0.780
	FC2	0.888				
	FC3	0.891				
	FC4	0.883				
HM	HM1	0.882	0.878	0.879	0.925	0.804
	HM2	0.917				
	HM3	0.890				
HT	HT1	0.889	0.868	0.872	0.919	0.791
	HT2	0.899				
	HT3	0.881				
PE	PE1	0.907	0.948	0.949	0.960	0.828
	PE2	0.910				
	PE3	0.921				
	PE4	0.915				
	PE5	0.896				
PI	PI1	0.872	0.851	0.853	0.909	0.770
	PI2	0.891				
	PI3	0.869				
PLO	PLO1	0.851	0.885	0.886	0.920	0.743
	PLO2	0.871				
	PLO3	0.868				
	PLO4	0.857				
PT	PT1	0.867	0.905	0.906	0.934	0.779
	PT2	0.906				
	PT3	0.910				
	PT4	0.846				
SI	SI1	0.820	0.875	0.880	0.908	0.665
	SI2	0.843				
	SI3	0.812				
	SI4	0.804				
	SI5	0.797				
Use	USE1	0.867	0.911	0.912	0.937	0.789
	USE2	0.908				
	USE3	0.899				
	USE4	0.879				

PE, performance expectancy; PT, perceived trust; EE, effort expectancy; SI, social influence; FC, facilitating condition; HM, hedonic motivation; PI, personal innovativeness; HT, habit; BI, behavioural intention; PLO, perceived learning opportunity; USE, use of GenAI; AVE, average variance extracted; VIF, variance inflation factor; CR, composite reliability; rho_a Dijkstra and Henseler’s Rho, rho_c, composite reliability.

**Figure 2:**
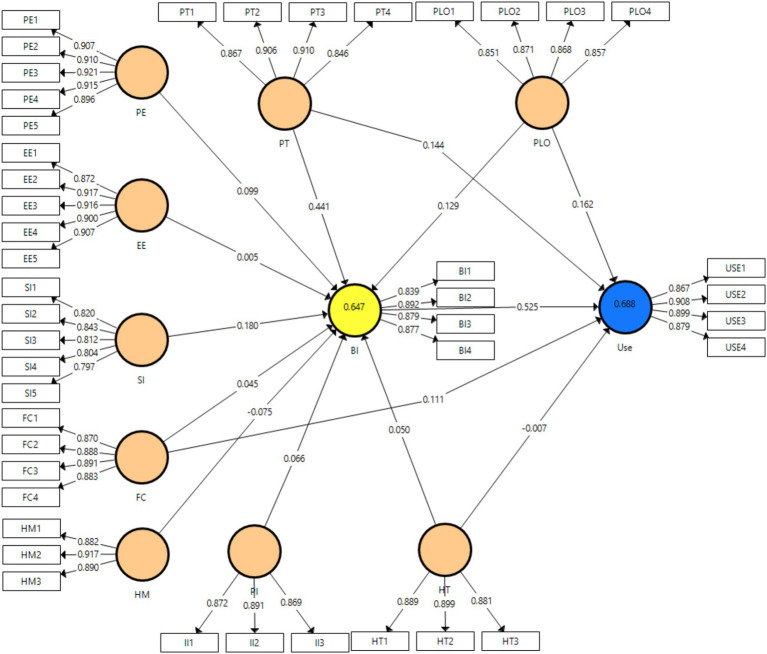
Algorithm results of PLS-SEM.

Construct reliability was further confirmed through composite reliability (CR) scores, which ranged between 0.908 and 0.960, all exceeding the recommended minimum of 0.70 (Hair et al., 2019). Convergent validity was equally established, as average variance extracted (AVE) values for every construct went beyond the 0.50 benchmark (see Table 1), in line with the standard set by Hair et al. (2022). Each measurement item also demonstrated a strong and clear loading on its intended construct, further strengthening the overall quality of the measurement model. Figure 2 presents the full output of the PLS-SEM algorithm.

### 4.4 Discriminant validity

Two established approaches were used to evaluate discriminant validity, namely the Fornell-Larcker criterion and the Heterotrait-Monotrait (HTMT) ratio, both of which yielded acceptable outcomes. Under the Fornell-Larcker criterion, a construct is said to demonstrate discriminant validity when it shares a greater proportion of variance with its own indicators than with any other construct in the model (Fornell and Larcker, 1981). Evidence of this was observed in the diagonal values recorded for constructs such as EE (0.903) and PE (0.910), as shown in Table 2, both of which were higher than their corresponding inter-construct correlations, confirming that this criterion was satisfied.

**Table 2:** Fornell and Larcker criterion.

Fornell and Larcker criterion											
Constructs	BI	EE	FC	HM	HT	PE	PI	PLO	PT	SI	Use
BI	0.872										
EE	0.603	0.903									
FC	0.614	0.594	0.883								
HM	0.593	0.633	0.776	0.896							
HT	0.610	0.562	0.660	0.687	0.890						
PE	0.602	0.859	0.565	0.581	0.556	0.910					
PI	0.640	0.597	0.685	0.743	0.771	0.577	0.877				
PLO	0.622	0.575	0.594	0.650	0.611	0.541	0.632	0.862			
PT	0.752	0.644	0.625	0.630	0.617	0.627	0.687	0.612	0.883		
SI	0.650	0.603	0.765	0.707	0.675	0.580	0.644	0.648	0.607	0.815	
Use	0.798	0.566	0.615	0.607	0.574	0.584	0.642	0.639	0.703	0.709	0.888

An examination of the HTMT ratio provided additional support, with the majority of values remaining below the widely accepted threshold of 0.90 (see Table 3) (Hair et al., 2019; Henseler et al., 2015). Collectively, the results from both methods confirm that each construct within the model is conceptually distinct and accurately captured by its measurement items.

**Table 3:** HTMT ratio criterion.

HTMT ratio											
Constructs	BI	EE	FC	HM	HT	PE	PI	PLO	PT	SI	Use
BI											
EE	0.658										
FC	0.681	0.642									
HM	0.669	0.696	0.866								
HT	0.689	0.618	0.743	0.787							
PE	0.653	0.806	0.608	0.637	0.611						
PI	0.731	0.667	0.779	0.860	0.894	0.642					
PLO	0.698	0.628	0.660	0.736	0.696	0.590	0.729				
PT	0.835	0.695	0.688	0.706	0.694	0.675	0.783	0.682			
SI	0.728	0.663	0.867	0.808	0.774	0.639	0.749	0.732	0.678		
Use	0.879	0.610	0.677	0.679	0.643	0.625	0.729	0.709	0.772	0.783	

### 4.5 Structural model

Bootstrapping, a non-parametric resampling approach, was adopted to evaluate all 14 hypotheses proposed in this study (Streukens and Leroi-Werelds, 2016). Prior to path testing, a multicollinearity check was conducted, and all VIF values were found to be under 5.0, ruling out any multicollinearity concerns in the dataset (Hair et al., 2019; Sarstedt et al., 2023). The model also demonstrated a satisfactory level of overall fit, as evidenced by an SRMR value of 0.046 (Ringle et al., 2023). Following this, a bootstrapping run of 10,000 resamples was executed to determine the significance of each structural path, as well as to compute explained variance (R^2^), effect sizes (f^2^), and predictive relevance (Q^2^). The findings related to the direct hypotheses are summarised in Table 4.

**Table 4:** Structural model.

Hypotheses	Path	β	M	SD	T-values	*p*-values	VIF	f^2^	5.0%	95.0%	Rks
H1	BI - > Use	0.525	0.524	0.043	12.356	0.000	2.689	0.329	0.455	0.596	SP
H2	EE - > BI	0.005	0.004	0.054	0.085	0.466	4.388	0.000	−0.085	0.091	NS
H3	FC - > BI	0.045	0.046	0.050	0.896	0.185	3.429	0.002	−0.039	0.127	NS
H4	FC - > Use	0.111	0.111	0.040	2.757	0.003	2.186	0.018	0.046	0.179	SP
H5	HM - > BI	−0.075	−0.074	0.050	1.505	0.066	3.552	0.004	−0.157	0.007	NS
H6	HT - > BI	0.050	0.051	0.049	1.034	0.151	2.952	0.002	−0.029	0.132	NS
H7	HT - > Use	−0.007	−0.006	0.038	0.185	0.427	2.203	0.000	−0.071	0.055	NS
H8	PE - > BI	0.099	0.100	0.050	1.988	0.023	4.018	0.007	0.016	0.178	SP
H9	PI - > BI	0.066	0.065	0.049	1.343	0.090	3.516	0.003	−0.014	0.147	NS
H10	PLO - > BI	0.129	0.128	0.037	3.448	0.000	2.216	0.021	0.068	0.191	SP
H11	PLO - > Use	0.162	0.162	0.033	4.968	0.000	2.029	0.042	0.108	0.216	SP
H12	PT - > BI	0.441	0.440	0.040	11.054	0.000	2.464	0.224	0.377	0.509	SP
H13	PT - > Use	0.144	0.144	0.038	3.810	0.000	2.710	0.025	0.082	0.206	SP
H14	SI - > BI	0.180	0.180	0.047	3.838	0.000	3.086	0.030	0.103	0.258	SP
Predicted Variables		R^2^	Q^2^								
BI		0.647	0.485								
USE		0.688	0.536								

β, path coefficient; f^2^, effect size; R^2^, R-square; Q^2^, predictive relevance; M, sample mean; Rks, remarks; SP supported, and NS, not supported; PE, performance expectancy; PT, perceived trust; EE, effort expectancy; SI, social influence; FC, facilitating condition; HM, hedonic motivation; PI, personal innovativeness; HT, habit; BI, behavioural intention; PLO, perceived learning opportunity; USE, use of GenAI; VIF, variance inflation factor; Adjusted R-square (BI), 0.643, and Adjusted R-square (USE), 0.686.

To assess the explanatory power of the model, the coefficient of determination (R^2^) was examined. R^2^ quantifies the proportion of variance in an endogenous construct that its predictor variables are able to account for (Hair et al., 2021). Following the classification by Hair et al. (2011), whereby R^2^ values of 0.75, 0.50, and 0.25 denote substantial, moderate, and weak explanatory power respectively, the present study recorded an R^2^ of 0.647, meaning that PE, PLO, PT, and SI jointly explained 64.7% of the variance in BI. In a similar manner, BI, FC, PLO, and PT collectively accounted for 68.8% of the variance in USE (R^2^ = 0.688).

**Figure 3:**
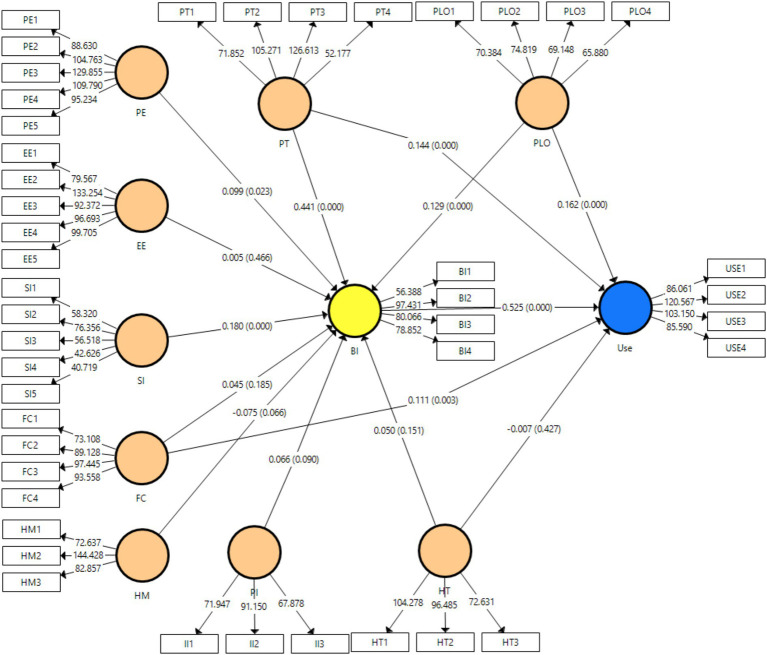
Bootstrapping results of PLS-SEM.

Given that R^2^ only measures how well the model fits within the sample and offers no indication of its predictive accuracy beyond it (Chin et al., 2020; Hair and Sarstedt, 2021), Q^2^ values were additionally calculated to assess out-of-sample predictive relevance. Drawing on the benchmarks provided by Hair et al. (2017), where Q^2^ values of 0.02, 0.15, and 0.35 indicate small, medium, and large predictive relevance respectively, the study obtained Q^2^ values of 0.485 for BI and 0.536 for USE. These figures point to a high level of predictive relevance for both constructs. Figure 3 illustrates the full bootstrapping output from the PLS-SEM analysis.

Beginning with the relationship between behavioural intention (BI) and system use, H_1_ was confirmed, as the path coefficient yielded a notably strong and statistically significant result (*β* = 0.525, *t* = 12.356, *p* < 0.001, f^2^ = 0.329). The remaining hypotheses were tested in sequence, with several failing to reach statistical significance. Specifically, the path linking effort expectancy (EE) to BI produced a negligible and non-significant result (*β* = 0.005, *t* = 0.085, *p* > 0.05), leaving H_2_ unsupported. Likewise, facilitating conditions (FC) showed no statistically significant bearing on BI (*β* = 0.045, *t* = 0.896, *p* > 0.05, f^2^ = 0.002), and H_3_ was consequently rejected. Interestingly, however, FC demonstrated a statistically significant positive effect on actual use (H_4_: *β* = 0.111, *t* = 2.757, *p* < 0.01, f^2^ = 0.018), and H_4_ was therefore accepted.

Among the motivational and habitual constructs, neither hedonic motivation (HM) nor habit (HT) emerged as significant predictors. HM failed to produce a significant effect on BI (H_5_: *β* = −0.075, *t* = 1.505, *p* > 0.05, f^2^ = 0.004), resulting in H_5_ being rejected. HT similarly showed no significant effect on BI (H_6_: *β* = 0.050, *t* = 1.034, *p* > 0.05, f^2^ = 0.002) or on actual use (H_7_: *β* = −0.007, *t* = 0.185, *p* > 0.05, f^2^ = 0.000), with both H_6_ and H_7_ left unsupported.

Turning to the supported hypotheses, performance expectancy (PE) registered a statistically significant positive effect on BI (H_8_: *β* = 0.099, *t* = 1.988, *p* < 0.05, f^2^ = 0.007), confirming H_8_. Although personal innovativeness (PI) pointed in a positive direction, its effect on BI fell short of statistical significance (H_9_: *β* = 0.066, *t* = 1.343, *p* > 0.05, f^2^ = 0.003), and H_9_ was not accepted. Perceived learning opportunity (PLO) proved to be a reliable predictor of both BI and actual use. Its positive effect on BI was statistically significant (H_10_: *β* = 0.129, *t* = 3.448, *p* < 0.001, f^2^ = 0.021), supporting H_10_, and its effect on actual use was equally strong and significant (H_11_: *β* = 0.162, *t* = 4.968, *p* < 0.001, f^2^ = 0.042), confirming H_11_.

Perceived trust (PT) stood out as the most influential construct in the model, recording the strongest positive effect on BI (H_12_: *β* = 0.441, *t* = 11.054, *p* < 0.001, f^2^ = 0.224) and a significant positive effect on actual use (H_13_: *β* = 0.144, *t* = 3.810, *p* < 0.001, f^2^ = 0.025), with both H_12_ and H_13_ receiving support. Rounding off the findings, social influence (SI) was confirmed as a significant positive predictor of BI (H_14_: *β* = 0.180, *t* = 3.838, *p* < 0.001, f^2^ = 0.030), lending support to H_14_.

### 4.6 Asymmetric analysis (fsQCA)

The study employed fsQCA (version 4.0) to investigate how combinations of factors influence preservice teachers’ use of GenAI as instructional media. This approach was chosen because it addresses the limitations of PLS-SEM in explaining complex relationships among variables. FsQCA examines configurations of conditions rather than individual effects, highlighting how multiple factors jointly shape GenAI Behavioural Intention and GenAI Use (Fiss, 2011). To complement this, a necessity analysis was conducted to identify essential predictors of both GenAI Behavioural Intention and Use (Dul, 2016). A condition is regarded as necessary when it satisfies the minimum consistency (≥ 0.8) and coverage (and ≥ 0.2) values (Ragin, 2008). For necessary antecedents, both consistency and coverage must exceed 0.9 (Dul, 2016). The fsQCA results revealed several sufficient configurations (see Appendix B) that explain high CBI among preservice teachers. Three main patterns emerged. The first reflects comprehensive competence and engagement, where combinations of CEE, CFC, CHM, CHT, CPE, and CPT jointly enhance behavioural intention. This suggests that strong support systems, relevant training, and perceived usefulness collectively strengthen teachers’ intention to use GenAI. The second pattern represents minimal resource and adaptive motivation, showing that even with limited CFC, CHM, and CHT, high CBI can occur when preservice teachers exhibit personal efficacy and intrinsic motivation. The third pattern, selective interaction and conditional commitment, highlights context-dependent combinations in which CPI, CPLO, and CPT contribute variably depending on CFC, CHM, and CHT. Overall solution coverage (0.9107) and consistency (0.8579) values indicate that high behavioural intention arises from multiple, complementary pathways rather than a single dominant condition. The necessary condition results highlighted that none of the predictors are necessary conditions for high BI (see Table 5).

**Table 5:** Analysis of necessary conditions for high and low GenAI Behavioural Intention (CBI).

Antecedents	Consistency	Coverage
CEE	0.861	0.861
CFC	0.870	0.871
CHM	0.865	0.866
CHT	0.862	0.867
CPE	0.863	0.864
CPI	0.874	0.876
CPLO	0.869	0.871
CPT	0.893	0.888
CSI	0.868	0.873
~CEE	0.857	0.856
~CFC	0.867	0.866
~CHM	0.862	0.861
~CHT	0.863	0.858
~CPE	0.860	0.858
~CPI	0.872	0.870
~CPLO	0.867	0.865
~CPT	0.884	0.889
~CSI	0.869	0.864

None of the antecedents are identified as necessary predictors of GenAI Behavioural Intention (CBI).

The fsQCA results revealed five sufficient configurations predicting high GenAI use (CUse) among preservice teachers (see Table 6). Individual core conditions such as CPLO (M1), CFC (M2), and CPT (M3) independently contributed to high CUse, reflecting the critical roles of learning orientation, facilitating conditions, and training. Additionally, two interactional pathways emerged: CHT combined with low CBI (M4) and low CHT combined with high CBI (M5), indicating that either strong technological competence or high behavioural intention can independently sustain GenAI use. The model showed high overall coverage (0.9792) and satisfactory consistency (0.7935), suggesting that multiple complementary routes effectively explain high CUse. Also, the necessary condition results revealed that BI is a necessary for high Use (see Table 7).

**Table 6:** Sufficient recipes to predict high CUse.

Configurations for CUse [CUse = f (CPLO, CFC, CHT, CPT, CBI)]					
Configuration Solution	M1: CPLO	M2: CFC	M3: CPT	M4: CHT* ~ CBI	M5: ~CHT*CBI
CPLO	●				
CFC		●			
CPT			●		
CHT				●	⊗
CBI				⊗	●
Raw coverage	0.875767	0.86913	0.884239	0.61322	0.646411
Unique coverage	0.015011	0.013628	0.013476	0.001257	0.006563
Consistency	0.877156	0.86926	0.878957	0.899299	0.940551
Solution coverage	0.979156				
Solution consistency	0.793508				

“●” indicates the presence of a condition; “⊗” indicates the negation of a condition; Blank cells represent “do not care” conditions; “~” denotes “negation”.

**Table 7:** Analysis of necessary conditions for high and low GenAI Use (CUse).

Antecedents	Consistency	Coverage
CPLO	0.876	0.877
CPT	0.884	0.879
CHT	0.860	0.864
CFC	0.869	0.869
CBI	*0.909*	0.908
~CPLO	0.873	0.872
~CPT	0.874	0.880
~CHT	0.861	0.856
~CFC	0.865	0.865
~CBI	*0.905*	0.906

Italicised values indicate necessary conditions (consistency > 0.90); CBI and ~CBI are identified as necessary predictors of GenAI Use (CUse).

## 5 Discussion

The findings confirmed behavioural intention as a strong driver of actual use, supporting (H_1_). This supports the widely held UTAUT2 view that of all the factors influencing behaviour, intention remains the one most closely and directly tied to what people actually do (Ajzen, 1991; Venkatesh et al., 2016). The result here is also supported by empirical work in Ghana and beyond showing that willingness to use AI leads to actual engagement (Dwivedi et al., 2021; Arthur et al., 2025; Basri, 2024). For example, Abreh et al. (2025) found Ghanaian STEM students’ intention to learn AI predicted their actual engagement behaviours, and Arthur et al. (2025) showed higher education students’ intention toward ChatGPT strongly predicted its use. However, Bayaga and du Plessis (2024) indicated that intention may not fully translate into use if institutional support is lacking. This implies that the existing relationship is moderated by other factors such as facilitating conditions as adequate support that enables actual use. Therefore, the result should be interpreted in context. Strong intention drives use, but sustained use may require ongoing support and positive experiences.

Conversely, effort expectancy did not significantly influence intention, rejecting (H_2_). This stands in contrast with studies; Teo et al. (2018) and Nguyen et al. (2024) who reported ease-of-use as a key adoption driver. However, the present finding is not entirely inconsistent with emerging evidence in educational technology adoption. For example, Bervell et al. (2022) and Zacharis and Nikolopoulou (2022) reported that effort expectancy did not significantly predict behavioural intention in technology-supported teaching contexts. These studies argued that when users already possess adequate digital competence or prior exposure to educational technologies, ease-of-use becomes less influential in shaping adoption decisions. A plausible interpretation is that Generative AI platforms have achieved an intuitive level of usability such that “effort” is no longer a differentiating factor for these preservice teachers. Abreh et al. (2025) revealed that effort expectancy mattered particularly for students with limited exposure. This suggests that the significance of effort expectancy may depend on users’ technological readiness and prior digital experience. In this case, the sample may already be digitally competent, making perceived ease less relevant in determining intention.

Similarly, facilitating conditions did not significantly predict intention, thus (H_3_) was rejected. This contradicts evidence that institutional support stimulates motivation to adopt AI (Abreh et al., 2025; Teo et al., 2018). But the present finding aligns with studies that reported weak or non-significant relationships between facilitating conditions and behavioural intention in educational technology contexts. Bervell et al. (2022) found that facilitating conditions did not directly predict teachers’ intention to use learning technologies, while Riady et al. (2022) also reported an insignificant relationship between facilitating conditions and behavioural intention among teachers. These contradictory findings suggest that the motivational role of facilitating conditions may vary depending on users’ prior technological exposure, perceived autonomy, and the accessibility of digital technologies outside institutional settings. This demonstrate that preservice teachers may desire to use GenAI irrespective of infrastructural constraints. Particularly when access to AI tools can be achieved through personal devices and publicly available digital platforms. This may reflect a growing personal drive to explore AI tools regardless of institutional readiness. The finding further suggests that facilitating conditions may not necessarily shape initial motivation when users already perceive the technology as professionally relevant or personally beneficial. Instead, their role may become more important during the implementation stage of adoption.

Yet facilitating conditions did significantly predict actual use, supporting (H_4_). This mirrors the argument by Cambra-Fierro et al. (2025) who stressed that structural readiness determines whether intention translates into behaviour. Similar findings were reported by Ng et al. (2023), who observed that institutional training and infrastructural support significantly influenced teachers’ actual integration of AI-supported assessment tools. In addition, Teo et al. (2018) found that while facilitating conditions did not always influence teachers’ intentions, they remained important for sustained and effective technology usage. Thus, FC appears to be more of a behavioural enabler than an attitudinal shaper in this context. This distinction is theoretically important because it suggests that preservice teachers may form favourable attitudes toward GenAI independently, but the actual execution of use still depends on the availability of institutional and technological support systems. Therefore, the transition from intention to actual GenAI use may be constrained more by structural realities than by motivational readiness alone.

Hedonic motivation did not significantly predict intention, rejecting (H_5_). This runs counter to earlier studies, including Wei et al. (2024) and Sallam et al. (2025) who argue that intrinsic enjoyment drives GenAI adoption. The result implies that preservice teachers may no longer view GenAI as a source of novelty or “fun”; instead, they may see it as a practical pedagogical tool. This interpretation is consistent with Zawacki-Richter et al. (2024), who observed that as AI technologies become more embedded in academic practice, utilitarian considerations increasingly outweigh affective enjoyment. Similarly, Zhang et al. (2025) noted that sustained educational use of AI depends more on perceived academic value than temporary excitement or curiosity. In other words, pleasure alone is insufficient to sustain adoption in this professionalising group. The finding therefore suggests that preservice teachers may approach GenAI less as an exploratory technology and more as a functional instructional resource linked to teaching effectiveness and professional preparation.

Similarly, habit showed no significant influence on either intention or actual use, rejecting (H_6_) and (H_7_). This contradicts Jang et al. (2021) and Tamilmani et al. (2019), who identified habit as a major driver of digital technology adoption. A logical explanation is that GenAI is still relatively new in teacher education contexts and has not yet reached a stage where it becomes automated or routine. Unlike conventional educational technologies such as learning management systems, GenAI applications are still evolving rapidly, which may limit repeated and stable usage patterns among preservice teachers. Thus, preservice teachers are still in a reflective and evaluative stage of adoption, not yet habitual. This interpretation aligns with Guan et al. (2025), who noted that although technologically experienced preservice teachers were more open to AI-supported pedagogy, sustained and routinized AI use had not yet fully developed. The finding therefore suggests that GenAI adoption among preservice teachers is currently driven more by conscious evaluation of pedagogical value than by automatic behavioural routines.

Supporting the UTAUT2 premise that belief in a technology’s ability to improve performance drives adoption, the significant positive effect of performance expectancy on behavioural intention (H_8_) confirms this theoretical expectation (Venkatesh et al., 2012). This corroborates with the findings of Dwivedi et al. (2021) and Wei et al. (2024), both found perceived usefulness to be a key driver of intentions to use AI-based systems in educational contexts. For preservice teachers, the practical benefit of GenAI, such as generating lesson ideas or improving instructional materials, appears to reinforce their motivation to engage with it. Thus, adoption is less about novelty and more about utility.

Personal innovativeness did not produce a meaningful or statistically significant impact on behavioural intention (H_9_). This contrasts with the expectations of Innovation Diffusion Theory (Rogers, 2003) and studies such as Al-Qaysi et al. (2023), where innovativeness significantly predicted early adoption of emerging technologies. The weak relationship here may reflect a contextual reality: preservice teachers might perceive GenAI as a mainstream educational tool rather than an innovation requiring risk-taking behaviour. Hence, innovativeness may have lost its explanatory power as AI use becomes normalized in educational practice.

The notable positive impact that perceived learning opportunity had on behavioural intention (H_10_) and actual use (H_11_) provides evidence that pedagogical usefulness remains central to GenAI engagement. This finding aligns with constructivist perspectives, which suggest that learners and teachers are more likely to adopt technologies that enhance knowledge acquisition, instructional effectiveness, and professional competence (Jonassen and Rohrer-Murphy, 1999). The finding also supports recent studies by Abreh et al. (2025) and Arthur et al. (2025), who reported that pre-service teachers’ perceptions of GenAI as a valuable learning resource significantly predicted both their behavioural intention and actual use. In this study, perceived learning opportunity appears to strengthen both intention and usage behaviour, suggesting that pre-service teachers are more likely to adopt GenAI when they perceive it as useful for improving teaching and learning outcomes.

Perceived trust showed the strongest direct predictor of behavioural intention (H_12_) and also recorded a notable influence on actual use (H_13_). These results confirm the growing body of evidence that trust is now a decisive component in the adoption AI (Sallam et al., 2025; Dwivedi et al., 2021). In teacher education, trust relates not only to data privacy or system reliability but also to epistemic trust, confidence in AI’s ability to generate accurate, ethical, and pedagogically sound content. The strong path coefficients underscore that even when GenAI tools are useful or easy to use, teachers will not commit to them unless they trust their outputs. This extends prior research by showing that trust functions both as a cognitive and behavioural trigger in AI-based learning environments.

Finally, the notable positive relationship between social influence on behavioural intention (H_14_) supports earlier findings from UTAUT-based studies underscored the part played by social norms in influencing how individuals adopt technology (Venkatesh et al., 2016; Teo et al., 2018). In the teacher preparation context, peer endorsement and institutional encouragement may signal legitimacy and relevance, thereby reinforcing adoption decisions. Similar observations were reported by Basri (2024), who found that peer discussions and faculty guidance significantly shaped preservice teachers’ openness toward AI tools. This suggests that social and institutional ecosystems remain crucial catalysts for building a positive adoption culture.

## 6 Conclusion

The study contributes a holistic view of the various drivers that shape GenAI adoption among preservice teachers. The results confirmed that adoption is not purely based on how easy or routine it is to use, but one grounded in perceptions of trust, pedagogical value, and performance utility. Key determinants such as perceived trust, performance expectancy, and perceived learning opportunity showed strong and significant effects on both intention and use. This indicates that when GenAI is seen as credible, helpful in their professional work, and suited to their learning requirements, preservice teachers are more likely to adopt it. Conversely, constructs often emphasised in mainstream technology adoption research such as effort expectancy, hedonic motivation, and habit, did not significantly predict intention or use in this teacher education context. This suggests that for future teachers, GenAI is evaluated more through professional lenses than casual or playful use. The study therefore provides empirical reinforcement to the argument that meaningful adoption in education requires more than technological novelty, it demands trustworthiness and added pedagogical value. Beyond these substantive findings, the study also makes an important methodological contribution to the educational technology literature through the integration of “PLS-SEM and fsQCA.” While PLS-SEM enabled the examination of linear and variance-based relationships among the study constructs, fsQCA provided additional insights into multiple configurational pathways leading to high behavioural intention and GenAI use. The combined application of these complementary analytical approaches offers a more nuanced understanding of technology adoption behaviour within teacher education and demonstrates the value of methodological pluralism in educational research contexts.

## 7 Implications

The findings present important implications at practical, policy, managerial, and theoretical levels, particularly in relation to how GenAI is taken up in teacher education settings. Practically, results indicate that strengthening preservice teachers’ trust in GenAI systems, and ensuring that such tools clearly demonstrate pedagogical value, is central to adoption. Teacher education programmes should therefore move beyond simple access and exposure, toward deliberate instructional modelling in which lecturers demonstrate how GenAI improves lesson quality, feedback generation, assessment support, and reflective practice. This has direct consequences for curriculum design: GenAI integration must be tied to demonstrable learning opportunities, rather than entertainment or habitual use.

At the managerial and institutional level, universities and Colleges of Education should develop structured policies and user-guidance frameworks around AI ethics, accuracy verification, academic integrity, and risk awareness. Because perceived trust and perceived learning opportunity were major drivers of intention and actual use, institutional efforts must prioritise transparency, quality assurance, and professional relevance. This includes curating AI tools with known reliability, providing institutional licenses, and offering professional development sessions that teach students how to evaluate GenAI outputs.

From a policy standpoint, regulating bodies, including national teaching councils and education ministries, should include AI competence standards in teacher education requirements. These standards should acknowledge that preservice teachers are more willing to take up GenAI when it demonstrably improves how they teach and learning effectiveness. Hence, policy direction should emphasise pedagogical integration rather than mere technological availability.

Theoretically, this study broadens the UTAUT2-based understanding of how GenAI is taken up within educational contexts by highlighting that some traditional constructs, such as habit, hedonic motivation, and effort expectancy, do not function strongly in this context. Instead, constructs that align with professional identity formation and evidence-based pedagogy, such as performance expectancy, perceived learning opportunity, and trust, are more salient. This calls for theoretical refinement in technology adoption models for teacher education, to better account for professional ethics, epistemic trust, and pedagogical utility in GenAI adoption.

## 8 Limitations and future research directions

Though this study offers novel empirical findings, it is imperative to recognise a number of limitations that come with it. First, the cross-sectional approach was adopted, which inherently constrains the ability to make definitive causal claims from the data, making it unclear whether behavioural intention will translate into sustained long-term use during practicum, internship, or early-career practice; therefore, longitudinal designs are required. Second, relying solely on participants’ self-reports poses a potential limitation, such as response-related and perceptual bias, suggesting that future research should integrate behavioural logs, usage analytics, or supervisor ratings to triangulate GenAI use. A third limitation is that the study took place within a specific cultural and institutional setting, thus replication across countries, institutional types, and different instructional traditions is necessary for broader generalisation. Fourth, while this study focused on UTAUT2 and pedagogically relevant variables, future research should examine additional constructs such as academic integrity concerns, critical AI literacy, epistemic trust, and teacher identity formation. Finally, the use of qualitative research methods could offer a richer and more detailed understanding of how student teachers decide when and in what ways to place their trust in GenAI, and how they negotiate ethical boundaries, thereby generating evidence-based design principles and training models that align GenAI adoption with professional and ethical teaching standards.

## 9 Recommendations

Based on these insights, several actionable implications emerge. First, teacher education institutions should embed GenAI literacy and critical AI evaluation skills in pedagogical training. This should focus on how GenAI can improve lesson design, feedback, assessment, and reflective practice, thereby strengthening perceptions of performance expectancy and learning opportunity. Second, institutions and AI tool providers must enhance transparent communication, accuracy assurances, and ethical usage guidelines to strengthen trust, given its powerful contribution to shaping both intention and actual use. Third, teacher education programmes should create structured peer-learning spaces and modelling practices where lecturers, mentors, and experienced peers demonstrate meaningful GenAI use, since social influence also meaningfully drives intention. Fourth, interventions should prioritise pedagogical use-cases of GenAI over entertainment uses, reinforcing that these tools are instructional partners rather than recreational gadgets. Finally, policymakers and curriculum bodies developing AI-in-education frameworks must move beyond access and infrastructure, and focus on trust calibration, AI risk literacy, academic integrity policies, and pedagogical integration standards. Collectively, these strategies will guide preservice teachers to not only adopt GenAI tools, but do so in informed, ethical, and pedagogically aligned ways that contribute to better teaching and learning outcomes.

## Data Availability

The raw data supporting the conclusions of this article will be made available by the authors, without undue reservation.
