# Supplementary material for: What drives preservice teachers’ use of generative AI as instructional media? A structural and configurational analysis
Source: Front Artif Intell. 2026 Jul 9;9:1853130. doi: 10.3389/frai.2026.1853130 (PMC13391880; doi:10.3389/frai.2026.1853130)
Supplement: Supplementary file 1 [file Supplementary_file_1.docx]

**Appendix A**

**Normality results**


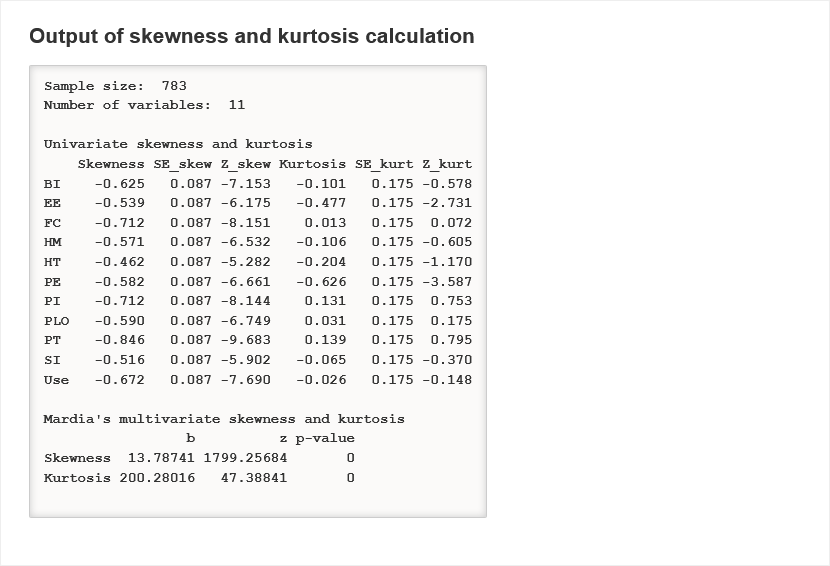


**Appendix B**

**Sufficient recipes to predict high CBI**

| Configurations for CBI = f (CEE, CFC, CHM, CHT, CPE, CPI, CPLO, CPT, CSI) | | | | | | |  |  |  |  |  |  |  |
| --- | --- | --- | --- | --- | --- | --- | --- | --- | --- | --- | --- | --- | --- |
|  |  |  |  |  |  |  |  |  |  |  |  |  |  |
| M | Configuration | CEE | CFC | CHM | CHT | CPE | CPI | CPLO | CPT | CSI | Raw coverage | Unique coverage | Consistency |
| M1 | CEE*CFC*CHM*CPE*CPI*CPT | ● | ● | ● |  | ● | ● |  | ● |  | 0.697587 | 0.001784 | 0.971177 |
| M2 | CEE*CFC*CHM*CPE*CPLO*CSI | ● | ● | ● |  | ● |  | ● |  | ● | 0.694271 | 0.004447 | 0.96985 |
| M3 | CEE*CFC*CHT*CPI*CPLO*CSI | ● | ● |  | ● |  | ● | ● |  | ● | 0.681155 | 0.000754 | 0.970398 |
| M4 | CFC*CHM*CHT*CPI*CPLO*CSI |  | ● | ● | ● |  | ● | ● |  | ● | 0.698843 | 0.008819 | 0.964558 |
| M5 | ~CFC*~CHM*~CHT*CPE*~CPI*~CPLO*~CPT |  | ⊗ | ⊗ | ⊗ | ● | ⊗ | ⊗ | ⊗ |  | 0.483342 | 0.00191 | 0.929998 |
| M6 | ~CEE*~CFC*~CHM*~CHT*~CPE*~CPI*CPLO | ⊗ | ⊗ | ⊗ | ⊗ | ⊗ | ⊗ | ● |  |  | 0.499497 | 0.001281 | 0.934605 |
| M7 | ~CEE*~CFC*~CHM*~CHT*~CPE*CPT*~CSI | ⊗ | ⊗ | ⊗ | ⊗ | ⊗ |  |  | ● | ⊗ | 0.493969 | 0.002538 | 0.951597 |
| M8 | ~CEE*~CHM*~CPE*~CPI*~CPLO*~CPT*CSI | ⊗ |  | ⊗ |  | ⊗ | ⊗ | ⊗ | ⊗ | ● | 0.493868 | 0.000402 | 0.94314 |
| M9 | ~CEE*CFC*CHM*~CHT*~CPE*~CPI*~CPT | ⊗ | ● | ● | ⊗ | ⊗ | ⊗ | ⊗ | ⊗ |  | 0.487135 | 0 | 0.945386 |
| M10 | ~CHM*~CHT*~CPE*CPI*~CPLO*CPT*~CSI |  |  | ⊗ | ⊗ | ⊗ | ● | ⊗ | ● | ⊗ | 0.481256 | 0.000503 | 0.971643 |
| M11 | ~CEE*~CHM*~CHT*CPI*~CPLO*CPT*~CSI | ⊗ |  | ⊗ | ⊗ |  | ● | ⊗ | ● | ⊗ | 0.482562 | 0.000402 | 0.970392 |
| M12 | CEE*~CFC*~CHM*~CHT*CPE*CPT*~CSI | ● | ⊗ | ⊗ | ⊗ | ● |  |  | ● | ⊗ | 0.492965 | 0.002186 | 0.971672 |
| M13 | ~CFC*~CHM*CPE*~CPI*CPLO*CPT*~CSI |  | ⊗ | ⊗ |  | ● | ⊗ | ● | ● | ⊗ | 0.480476 | 0.000075 | 0.97224 |
| M14 | CEE*~CHM*~CHT*CPE*CPLO*CPT*~CSI | ● |  | ⊗ | ⊗ | ● |  | ● | ● | ⊗ | 0.485502 | 0.001457 | 0.979868 |
| M15 | CEE*~CFC*~CHT*CPE*~CPI*CPLO*CPT | ● | ⊗ |  | ⊗ | ● | ⊗ | ● | ● |  | 0.49103 | 0.000804 | 0.978863 |
| M16 | CEE*CFC*CHM*~CHT*CPE*CPI*CPLO | ● | ● | ● | ⊗ | ● | ● | ● |  |  | 0.517312 | 0.000402 | 0.979356 |
| M17 | CEE*CFC*CHT*CPE*CPI*~CPLO*CPT | ● | ● |  | ● | ● | ● | ⊗ | ● |  | 0.522938 | 0.000226 | 0.983183 |
| M18 | CFC*CHT*CPE*CPI*CPLO*~CPT*CSI |  | ● |  | ● | ● | ● | ● | ⊗ | ● | 0.524548 | 0 | 0.978166 |
| M19 | CEE*CHM*CHT*CPE*CPI*CPLO*CPT | ● |  | ● | ● | ● | ● | ● | ● |  | 0.677787 | 0.003392 | 0.978455 |
| M20 | CEE*CHM*CHT*CPE*CPI*CPLO*CSI | ● |  | ● | ● | ● | ● | ● |  | ● | 0.675325 | 0.001432 | 0.975892 |
| M21 | CEE*CFC*CHM*CHT*CPE*CPT*CSI | ● | ● | ● | ● | ● |  |  | ● | ● | 0.6752 | 0.001357 | 0.977947 |
| M22 | CEE*CFC*CHT*CPE*CPLO*CPT*CSI | ● | ● |  | ● | ● |  | ● | ● | ● | 0.667386 | 0.000527 | 0.977622 |
| M23 | CEE*~CHM*~CHT*~CPE*~CPI*~CPLO*~CPT*~CSI | ● |  | ⊗ | ⊗ | ⊗ | ⊗ | ⊗ | ⊗ | ⊗ | 0.469346 | 0 | 0.932834 |
| M24 | ~CEE*~CFC*~CHM*CHT*~CPE*~CPI*~CPLO*~CSI | ⊗ | ⊗ | ⊗ | ● | ⊗ | ⊗ | ⊗ | ⊗ | ⊗ | 0.486405 | 0.00103 | 0.936211 |
| M25 | ~CEE*~CFC*~CHM*~CHT*~CPE*CPI*~CPLO*~CSI | ⊗ | ⊗ | ⊗ | ⊗ | ⊗ | ● | ⊗ | ⊗ | ⊗ | 0.484773 | 0.001131 | 0.946062 |
| M26 | CEE*~CFC*CHM*~CHT*~CPE*~CPLO*~CPT*~CSI | ● | ⊗ | ● | ⊗ | ⊗ | ⊗ | ⊗ | ⊗ | ⊗ | 0.461205 | 0 | 0.960443 |
| M27 | ~CEE*CFC*CHM*~CHT*~CPE*~CPLO*~CPT*~CSI | ⊗ | ● | ● | ⊗ | ⊗ | ⊗ | ⊗ | ⊗ | ⊗ | 0.470049 | 0 | 0.953954 |
| M28 | ~CEE*CFC*~CHT*~CPE*~CPI*CPLO*~CPT*~CSI | ⊗ | ● |  | ⊗ | ⊗ | ⊗ | ● | ⊗ | ⊗ | 0.469647 | 0 | 0.960484 |
| M29 | ~CEE*CFC*~CHM*~CHT*~CPE*~CPI*~CPLO*CSI | ⊗ | ● | ⊗ | ⊗ | ⊗ | ⊗ | ⊗ | ⊗ | ● | 0.483718 | 0.00098 | 0.957953 |
| M30 | CEE*~CFC*~CHM*CHT*CPI*~CPLO*~CPT*~CSI | ● | ⊗ | ⊗ | ● |  | ● | ⊗ | ⊗ | ⊗ | 0.463868 | 0 | 0.968676 |
| M31 | ~CEE*CFC*~CHM*CPE*~CPI*CPLO*~CPT*~CSI | ⊗ | ● | ⊗ |  | ● | ⊗ | ● | ⊗ | ⊗ | 0.452461 | 0.000603 | 0.974933 |
| M32 | CEE*CFC*CHM*~CHT*CPE*~CPI*~CPLO*~CPT | ● | ● | ● | ⊗ | ● | ⊗ | ⊗ | ⊗ | ⊗ | 0.467035 | 0 | 0.972379 |
| M33 | CEE*~CFC*~CHM*CHT*CPE*CPI*~CPLO*~CPT | ● | ⊗ | ⊗ | ● | ● | ● | ⊗ | ⊗ | ⊗ | 0.471155 | 0 | 0.974332 |
| M34 | CEE*~CFC*CHT*CPE*~CPI*CPLO*~CPT*~CSI | ● | ⊗ |  | ● | ● | ⊗ | ● | ⊗ | ⊗ | 0.465426 | 0.000301 | 0.975563 |
| M35 | CEE*~CFC*~CHM*CPE*CPI*CPLO*~CPT*~CSI | ● | ⊗ | ⊗ |  | ● | ● | ● | ⊗ | ⊗ | 0.467185 | 0.000603 | 0.979766 |
| M36 | CEE*CFC*CHM*~CPE*~CPI*~CPLO*CPT*~CSI | ● | ● | ● |  | ⊗ | ⊗ | ⊗ | ● | ⊗ | 0.465124 | 0 | 0.98353 |
| M37 | CEE*CFC*~CHM*~CHT*CPE*~CPI*~CPLO*CPT | ● | ● | ⊗ | ⊗ | ● | ⊗ | ⊗ | ● |  | 0.47309 | 0.000352 | 0.983032 |
| M38 | CEE*~CFC*~CHT*CPE*CPI*~CPLO*CPT*~CSI | ● | ⊗ |  | ⊗ | ● | ● | ⊗ | ● | ⊗ | 0.470226 | 0.000327 | 0.982518 |
| M39 | CEE*~CFC*~CHM*CPE*CPI*~CPLO*CPT*~CSI | ● | ⊗ | ⊗ |  | ● | ● | ⊗ | ● | ⊗ | 0.476909 | 0.000854 | 0.982708 |
| M40 | ~CEE*CFC*~CHT*~CPE*CPI*CPLO*CPT*~CSI | ⊗ | ● |  | ⊗ | ⊗ | ● | ● | ● | ⊗ | 0.470829 | 0.000151 | 0.98533 |
| M41 | ~CFC*~CHM*CHT*~CPE*CPI*CPLO*CPT*~CSI |  | ⊗ | ⊗ | ● | ⊗ | ● | ● | ● | ⊗ | 0.473868 | 0 | 0.983213 |
| M42 | ~CEE*CFC*CHM*~CHT*~CPE*~CPI*CPLO*CSI | ⊗ | ● | ● | ⊗ | ⊗ | ⊗ | ● | ⊗ | ● | 0.483818 | 0.001181 | 0.969489 |
| M43 | ~CEE*~CFC*~CHM*CHT*CPI*~CPLO*CPT*CSI | ⊗ | ⊗ | ⊗ | ● |  | ● | ⊗ | ● | ● | 0.482536 | 0.000653 | 0.987556 |
| M44 | ~CEE*CFC*CHM*CHT*CPE*CPI*~CPLO*~CPT | ⊗ | ● | ● | ● | ● | ● | ⊗ | ⊗ | ⊗ | 0.472185 | 0.000201 | 0.980487 |
| M45 | ~CEE*CFC*CHM*CHT*~CPE*CPI*~CPLO*CPT | ⊗ | ● | ● | ● | ⊗ | ● | ⊗ | ● |  | 0.489546 | 0.001206 | 0.986981 |
| M46 | ~CEE*CFC*CHM*~CHT*CPI*CPLO*CPT*~CSI | ⊗ | ● | ● | ⊗ |  | ● | ● | ● | ⊗ | 0.471181 | 0.000452 | 0.984875 |
| M47 | ~CEE*CFC*~CHM*CHT*~CPE*CPI*CPLO*CPT | ⊗ | ● | ⊗ | ● | ⊗ | ● | ● | ● |  | 0.482662 | 0.000904 | 0.986595 |
| M48 | ~CEE*CFC*CHM*~CHT*CPE*~CPLO*CPT*CSI | ⊗ | ● | ● | ⊗ | ● | ⊗ | ⊗ | ● | ● | 0.468844 | 0.001055 | 0.984956 |
| M49 | ~CEE*~CFC*CHM*~CPE*CPI*CPLO*CPT*CSI | ⊗ | ⊗ | ● | ⊗ | ⊗ | ● | ● | ● | ● | 0.489169 | 0.000653 | 0.988324 |
| M50 | ~CEE*CFC*CHT*CPE*CPI*CPLO*CPT*~CSI | ⊗ | ● |  | ● | ● | ● | ● | ● | ⊗ | 0.470351 | 0.001206 | 0.985782 |
| M51 | ~CEE*CFC*~CHM*CHT*~CPE*CPI*~CPLO*~CPT*~CSI | ⊗ | ● | ⊗ | ● | ⊗ | ● | ⊗ | ⊗ | ⊗ | 0.459521 | 0.000176 | 0.976298 |
| M52 | `CEE*~CFC*CHM*CHT*CPE*~CPI*~CPLO*~CPT |  |  |  |  |  |  |  |  |  |  |  |  |

Note: “● = indicates the presence of a condition; ⊗ = indicates the negation of a condition; Blank cells represent = don’t care conditions; ~ = negation”
